# Simulation of the θ′ Precipitation Process with Interfacial Anisotropy Effects in Al-Cu Alloys

**DOI:** 10.3390/ma14051280

**Published:** 2021-03-08

**Authors:** Na Ta, Muhammad Umer Bilal, Ines Häusler, Alaukik Saxena, Yueh-Yu Lin, Felix Schleifer, Michael Fleck, Uwe Glatzel, Birgit Skrotzki, Reza Darvishi Kamachali

**Affiliations:** 1 Max-Planck-Institut für Eisenforschung GmbH, Max-Planck-Straße 1, 40237 Düsseldorf, Germany; tagetacpu@163.com (N.T.); saxenaalaukik93@gmail.com (A.S.); 2 School of Material Science and Engineering, University of Science & Technology Beijing, Beijing 100083, China; 3 Helmholtz-Zentrum Geesthacht GmbH, Max-Planck-Straße 1, 21502 Geesthacht, Germany; muhammad.bilal@hzg.de; 4 Institute of Optics and Atomic Physics, Technical University Berlin, 10623 Berlin, Germany; haeusler@tu-berlin.de; 5 Metals and Alloys, University of Bayreuth, Prof.-Rüdiger-Bormann-Straße 1, 95447 Bayreuth, Germany; yueh-yu.lin@uni-bayreuth.de (Y.-Y.L.); felix.schleifer@uni-bayreuth.de (F.S.); michael.fleck@uni-bayreuth.de (M.F.); uwe.glatzel@uni-bayreuth.de (U.G.); 6 Federal Institute for Materials Research and Testing (BAM), 12205 Berlin, Germany; birgit.skrotzki@bam.de

**Keywords:** phase-field simulation, interfacial anisotropy, chemo-mechanical coupling, precipitation, elasticity, θ′-(Al_2_Cu) precipitate phase, aging

## Abstract

The effects of anisotropic interfacial properties and heterogeneous elasticity on the growth and ripening of plate-like θ′-phase (Al_2_Cu) in Al-1.69 at.% Cu alloy are studied. Multi-phase-field simulations are conducted and discussed in comparison with aging experiments. The precipitate/matrix interface is considered to be anisotropic in terms of its energy and mobility. We find that the additional incorporation of an anisotropic interfacial mobility in conjunction with the elastic anisotropy result in substantially larger aspect ratios of the precipitates closer to the experimental observations. The anisotropy of the interfacial energy shows comparably small effect on the precipitate’s aspect ratio but changes the interface’s shape at the rim. The effect of the chemo-mechanical coupling, i.e., the composition dependence of the elastic constants, is studied as well. We show that the inverse ripening phenomenon, recently evidenced for δ’ precipitates in Al-Li alloys (Park et al. Sci. Rep. 2019, 9, 3981), does not establish for the θ′ precipitates. This is because of the anisotropic stress fields built around the θ′ precipitates, stemming from the precipitate’s shape and the interaction among different variants of the θ′ precipitate, that disturb the chemo-mechanical effects. These results show that the chemo-mechanical effects on the precipitation ripening strongly depend on the degree of sphericity and elastic isotropy of the precipitate and matrix phases.

## 1. Introduction

Understanding the precipitation process is of paramount importance for developing Al alloys. This requires a comprehensive consideration of several phenomena, including diffusion of solutes, misfit stresses generated by the solutes and precipitates, and the Gibbs-Thompson effect due to the small precipitates’ size. A key complicating factor is the mutual interplay between these phenomena, i.e., the chemo-mechanical coupling between diffusing solutes and the stress fields around a precipitate, amplified by the small size of the precipitates. The importance of the chemo-mechanical coupling has been discussed in various contexts [1,2,3,4,5,6]. Recent theoretical and experimental studies have revealed that the mutual interactions between stress fields and solutal gradients at the precipitation front can alter precipitates’ growth and ripening kinetics [7,8,9,10]. The origin of the chemo-mechanical coupling effect on the precipitate ripening can be traced back to its ability for partially stabilizing solute atoms in the matrix phase, above its equilibrium composition. Such stabilizing concept has the potential to address abnormal stability of core-shell precipitates as reported, for instance, in ternary Al-Li-Sc alloys [11].

Experimental investigation of precipitate ripening has shown that the chemo-mechanical coupling effects can be very sensitive to the distribution of the stress fields around the precipitates [1]. This makes the shape of the precipitates very important. The main precipitate’s shape-controlling factors are; (1) the anisotropic elasticity of the precipitate/matrix phases; and (2) the anisotropic properties of the interface between them. The mutual interactions among the precipitates can further influence the stress fields. In this work, we study the effects of the anisotropic interface and elastic properties on the growth and ripening of the θ′ phase in an Al-Cu alloy.

Various elements have been used to tailor Al properties via alloying for specific applications [12,13]. Cu is the main element in the age hardenable 2xxx alloy series which possesses a good combination of high strength and toughness. The binary Al-Cu alloy system has been well known for its interesting precipitation sequence. The θ-Al_2_Cu is the thermodynamically stable phase in this alloy. However, it is kinetically unfavorable for direct precipitation from the solid solution matrix, especially at temperatures below 250 °C. The sequence of transformations is [12]:

Al_ss_ → GP zones → θ″ → θ′ → θ

While nucleation of the θ′ precipitates from the supersaturated Al solid solution (Al_ss_) is a complicated process, it is generally accepted that initially Guinier-Preston (GP) zones (a monolayer of Cu atoms on the {100} plane of the Al-matrix) are formed. A transition phase θ″ (two layers of Cu atoms separated by three layers of Al atoms, i.e., Cu/Al/Al/Al/Cu) develops from the Guinier–Preston (GP) zones in a subsequent step. This is followed by formation of the θ′ precipitates which eventually are replaced by the equilibrium θ phase. The θ′ precipitates can nucleate either on GP zones, θ″ precipitates, or at defects, especially dislocations (heterogeneous nucleation). In contrast, θ precipitates often tend to nucleate at high angle grain boundaries (>10°) [14].

Experimental measurements show that peak hardening in Al-Cu alloys occurs in the temperature range of 190–230 °C. This is associated with the presence of the plate-like θ′ precipitates. The orientation relationship (100)_θ′_||(100)_Al_ and [100]_θ′_||[100]_Al_ results in three crystallographic variants of the θ′ phase [12,15,16]. The θ′ precipitates can effectively impede the motion of dislocations that also benefits from their plate-like morphology [17,18]. The probability of identifying optimal heat treatments, resulting into the best possible size and shape of precipitates, through experimental trial-and-error is slim and prohibitively expensive. Here, simulation tools can be used as virtual experiments to investigate the effects of different heat treatments on precipitation.

The θ′ precipitates possess a plate-shaped morphology with coherent broad faces of low interfacial energy (~0.17 J/m^2^) and semi-coherent interfaces around the rim [14] with higher interfacial energy (~0.52 J/m^2^) [19,20,21]. The equilibrium shape of the θ′ plates depends on two effects: (1) The anisotropy of the interfacial properties differing at the plate’s faces and rims; and (2) the strain energy which arises from differences in the elastic constants and molar volumes between the precipitate and matrix phases. The first effect has two aspects to it, i.e., the interfacial free energy and mobility. The free energy associated with formation of the interfaces between different phases can vary depending on their crystallographic orientations. In the case of θ′ precipitate, the interface at the rim has rather higher energies that, together with the higher local curvatures at the rim, tend to increase the sphericity of the rim. The anisotropy of the interface mobility, instead, is related to the mechanisms of attachment/detachment of the atoms across an interface. A ‘higher’ interfacial mobility means that the structural change and the attachment/detachment of the atoms across the interface occur ‘easier’ with either a lower energy barrier, a higher attachment/detachment frequency, or both. A ‘lower’ interface mobility means that the structural rearrangement at the interface may require higher driving forces and the interface migrates comparatively slower.

Analytical [22,23,24] and experimental [25,26,27,28] investigations showed that the physical mechanism for plate thickening of the θ′ phase is the motion of ledges across the broad face that need to nucleate in the first place, while along the θ′ rim atoms attach to the already existing ledges. This motivates that the interface mobility can be rather anisotropic. A recent theoretical approach on the growth of ellipsoidal precipitates was developed by Larouche [29]. This theory proposes that the subcritical growth regime occurring during the nucleation can be described as a one-stage process. The activation energy for this process is associated to the mobility of the interface. The experimental determination of this parameter is described by Heugue for the θ′-Al_2_Cu and θ-Al_2_Cu precipitates in a binary Al-3.5 wt.% Cu alloy [30]. These suggest that the influence of the interface mobility on the precipitate shape might need a more detailed investigation.

The simplest model for the evolution of the precipitation is to describe it by a single parameter, the volume fraction of precipitates *V_f_* for which a simple Johnson-Mehl-Avrami type approach might be chosen. The next level of complexity is to describe the precipitate microstructure by an average precipitate size, *R*, and number density, *N_v_*. The volume fraction can be calculated based on these two parameters and the precipitate shape [31]. However, these are not suitable for non-spherical precipitates. In the case of plate-like precipitates, the mobility depends clearly on the local atomic arrangement at the interfaces. Calculations of the interfacial mobility of the precipitate need to consider effects on an atomic scale that are currently being made using Molecular Dynamics (MD) simulations [32]. Wang et al. [33] have used Lattice Kinetic Monte Carlo (LKMC) approach to describe the precipitation of GP-zones in Al-Cu alloys. However, larger precipitate sizes and heat treatment timeframes, comparable to the aging experiments, are usually too expensive on the computational resources for existing atomistic simulation approaches [32].

Mesoscale approaches may seem more appealing to achieve larger length and time scales [34,35]. The meso-scale phase-field approach has also recently been employed to simulate shape formation of plate-like precipitates [36]. Li and Chen [37] simulated stress-oriented nucleation and growth of θ′ precipitates using two parabolic functions to describe the chemical free energies for the θ′- and α-phases. Based on first-principle calculations, Vaithyanathan et al. [14,38] also utilized parabolic functions to describe chemical free energies of the θ′ and α-phases, and studied the effect of elastic energy and interface anisotropy on the morphology and growth of θ′ precipitates. In the current study, we employ similar anisotropy functions but rather focus on separating the effects of anisotropic interfacial energy and mobility on the shape of the precipitate. We further study precipitation ripening in the presence of chemo-mechanical coupling, i.e., the composition dependence of the elastic constants in the matrix [1,9,10]. Experimental and large-scale multi-phase-field simulation studies are performed, considering θ′ (Al_2_Cu) precipitation in an Al-1.69 at.% Cu alloy at 453 K. We investigate the dominating factors controlling precipitation process and morphology, and how they affect the stress and concentration fields around the precipitates. The effects of stress fields on the precipitate ripening with and without the chemo-mechanical coupling effects are compared.

## 2. Model Descriptions

### Multi-Phase-Field Model

A multi-phase-field (MPF) model is used through the open-source microstructure simulation package OpenPhase [39,40]. On a given volume *Ω*, a general free energy density can be split into interfacial $f^{\mathrm{intf}}$, chemical $f^{\mathrm{chem}}$ and elastic $f^{\mathrm{elast}}$ contributions [41,42,43,44],

(1) $$F=\int_{\Omega }f^{\mathrm{intf}}+f^{\mathrm{chem}}+f^{\mathrm{elast}}\;dV$$

(2) $$f^{\mathrm{intf}}=\sum_{\alpha ,\;\beta =1}^{N}\frac{4\sigma_{\alpha \beta }}{\eta_{\alpha \beta }}\left\{-\frac{\eta_{\alpha \beta }^{2}}{\pi^{2}}\nabla \varphi_{\alpha }\cdot \nabla \varphi_{\beta }+\varphi_{\alpha }\varphi_{\beta }\right\}$$

(3) $$f^{\mathrm{chem}}=\sum_{\alpha =1}^{N}h\left(\varphi_{\alpha }\right)f_{\alpha }\left(c_{\alpha }^{i}\right)+{\tilde{\mu }}^{i}\left(c^{i}-\sum_{\alpha =1}^{N}\varphi_{\alpha }c_{\alpha }^{i}\right)$$

(4) $$f^{\mathrm{elast}}=\frac{1}{2}\sum_{\alpha =1}^{N}h\left(\varphi_{\alpha }\right)\left({\bar{\varepsilon }}_{\alpha }-{\bar{\varepsilon }}_{\alpha }^{*}\right){\bar{C}}_{\alpha }\left({\bar{\varepsilon }}_{\alpha }-{\bar{\varepsilon }}_{\alpha }^{*}\right)$$

where *N* is the local number of phases and the sum constraint $\sum_{\alpha =1}^{N}\varphi_{\alpha }=1$ is everywhere fulfilled. *φ*_α_ is the phase-field of α-phase, *σ*_αβ_ is the interfacial energy between the phases *α* and *β*, *η*_αβ_ is the interface width, *h*(*φ*_α_) is a weighting function and $f_{\alpha }\left(c_{\alpha }^{i}\right)$ is the bulk free energy density of the individual phase α, which depends on the phase concentrations $c_{\alpha }^{i}$. ${\tilde{\mu }}^{i}$ is the diffusion potential of component *i* introduced as a Lagrange multiplier to conserve the mass balance between the phases and we have $c^{i}=\sum_{\alpha =1}^{N}\varphi_{\alpha }c_{\alpha }^{i}=1$. ${\bar{\varepsilon }}_{\alpha }$ is the total strain tensor, ${\bar{\varepsilon }}_{\alpha }^{*}$ is the eigenstrain tensor, and ${\bar{C}}_{\alpha }$ is the elastic stiffness of the phase α. The equations of motion for the phase-field *φ*_α_, concentration *c^i^* and strain/stress ${\bar{\varepsilon }}_{\alpha }/{\bar{C}}_{\alpha }\left({\bar{\varepsilon }}_{\alpha }-{\bar{\varepsilon }}_{\alpha }^{*}\right)$ fields are given by [41,42,43,44],

(5) $${\dot{\varphi }}_{\alpha }=\;\sum_{\alpha =1}^{N}\mu_{\alpha \beta }\left\{\sigma_{\alpha \beta }\left[\nabla^{2}\varphi_{\alpha }-\nabla^{2}\varphi_{\beta }+\frac{\pi^{2}}{2\eta_{\alpha \beta }^{2}}\left(\varphi_{\alpha }-\varphi_{\beta }\right)\right]+\frac{\pi }{\eta_{\alpha \beta }}\sqrt{\varphi_{\alpha }\varphi_{\beta }}\Delta G_{\alpha \beta }\right\}$$

(6) $${\dot{c}}^{i}=\nabla \cdot \nabla \sum_{\alpha =1}^{N}\varphi_{\alpha }M_{\alpha }\nabla {\tilde{\mu }}_{\alpha }^{i}$$

(7) $$0=\nabla \sum_{\alpha =1}^{N}\varphi_{\alpha }{\bar{C}}_{\alpha }\left({\bar{\varepsilon }}_{\alpha }-{\bar{\varepsilon }}_{\alpha }^{*}\right)$$

where $\mu_{\alpha \beta }$ is the interfacial mobility and $\Delta G_{\alpha \beta }$ is the local deviation from thermodynamic equilibrium, consisting of the chemical $\Delta G_{\alpha \beta }^{\mathrm{chem}}$ and the elastic $\Delta G_{\alpha \beta }^{\mathrm{elast}}$ parts:

(8) $$\Delta G_{\alpha \beta }^{\mathrm{chem}}=-f_{\alpha }\left(c_{\alpha }^{i}\right)+f_{\beta }\left(c_{\beta }^{i}\right)+{\tilde{\mu }}^{i}\left(c_{\alpha }^{i}-c_{\beta }^{i}\right)$$

(9) $$\Delta G_{\alpha \beta }^{\mathrm{elast}}=\left({\bar{\varepsilon }}_{\alpha }^{*}-{\bar{\varepsilon }}_{\beta }^{*}\right){\bar{s}}_{\alpha }.$$

The chemical part $\Delta G_{\alpha \beta }^{\mathrm{chem}}$ can be linked to the CALPHAD (CALculation of PHAse Diagram) thermodynamic databases [45] and ${\bar{s}}_{\alpha }$ is the stress tensor subject to proper homogenization scheme [46]. $M_{\alpha }$ is the atomic mobility in phase α, and can be directly obtained from the CALPHAD mobility databases [47]. ${\tilde{\mu }}_{\alpha }^{i}$ is the diffusion potential of component *i* in phase α. The chemo-mechanical effects were introduced by considering a linear composition dependence of elastic constants as detailed in [10,39].

Comparing the plate-like morphology of θ′ precipitates in the experiments to our previous simulation results [48], it is clear that the simulated precipitates exhibit a pronounced ellipsoidal shape, deviating from the experimental observation. This can be due to the interfacial anisotropy effects that were not considered. In the present work, the interfacial anisotropy model based on the work of Ji et al. [49] has been considered. It is assumed that the interfacial mobility *μ* and energy *σ* depend on the local interface orientation according to $\mu =\mu_{0}\;I\left(\theta_{i}\right),$ and $\sigma =\sigma_{0}\;I\left(\theta_{i}\right)$, respectively. The anisotropy function $I\left(\theta_{i}\right)$ follows,

(10) $$I\left(\theta_{i}\right)=\left\{\begin{array}{c} \frac{1}{1+\beta }\left(1+\frac{\beta }{sin\phi_{0}}+\frac{\beta \;cos\phi_{0}}{sin\phi_{0}}\right)\;-\frac{\pi }{2}\leq \theta_{i}<-\frac{\pi }{2}+\phi_{0}\\ \frac{1}{1+\beta }\left(1+\alpha cos\theta_{i}\right)\;-\frac{\pi }{2}+\phi_{0}\leq \theta_{i}\leq \frac{\pi }{2}-\phi_{0}\\ \frac{1}{1+\beta }\left(1+\frac{\beta }{sin\phi_{0}}-\frac{\beta \cos\phi_{0}}{sin\phi_{0}}\right)\;\frac{\pi }{2}-\phi_{0}<\theta_{i}\leq \frac{\pi }{2} \end{array}\right.$$

where parameters $\mu_{0}$ and $\sigma_{0}$ correspond to mobility/energy of interfaces with an orientation angle equals to 0. *β* denotes the dimensionless anisotropy strength, which is determined as the relative difference between the mobility/energy value at interface orientation angles 0 and ±π/2 and, the interface orientation angle *θ_i_* relates to the phase-field variables and is calculated as:

(11) $$\theta_{i}=\sin^{-1}\left(\frac{e_{z}\nabla \varphi }{\left|\nabla \varphi \right|}\right).$$

**Figure 1:**
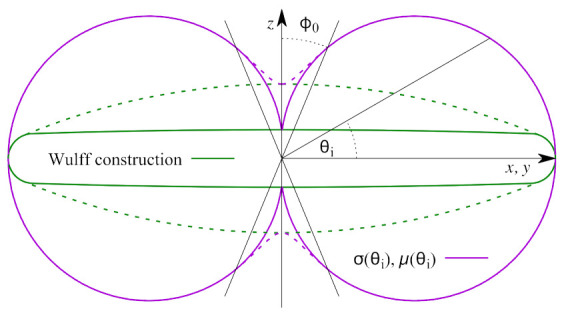
Parametric plot of the orientation dependence of the interface energy and mobility according to Equation (10) together with the parametric Wulff construction of the equilibrium shape of a particle.

In Figure 1, we plot the anisotropy function Equation (10) for an anisotropy strength *β* = 10. This function, originally proposed by Debierre et al. [50], has the advantage that regardless of the imposed anisotropy strength the Wulff-construction does not develop sharp corners, where certain high energy orientations are excluded. Generally, these corners or “ears” in the Wulff-construction should be avoided, as the phase-field evolution is ill-posed for these interface orientations [51,52]. To avoid a non-differential cusp in the anisotropy function for interface orientations close to ±π/2 a threshold angle *φ*_0_ has been defined [50]. In Figure 1, we compare the anisotropy function as well as the respective Wulff constructions for the cases *φ*_0_ = π/100 (solid lines) and *φ*_0_ = π/8 (dashed lines).

The maximum interfacial mobility is set as *µ*_max_ = *µ*_0_ = 1 × 10^−19^ m^4^/Js, according to the Equation (35) in Ref. [41], to guarantee the diffusion-controlled transformation. Thus the ratio between the horizontal (rims) and vertical (faces) growth direction can be obtained based on the corresponding anisotropy strength of interfacial mobility *β*_µ_ = 2000, *µ*_min_/*µ*_max_ = 1/(1 + *β*_µ_) = 1/2001 = 5 × 10^−4^_._ The semi-coherent interfacial energy *σ*_0_ = 0.245 J/m^2^, and the coherent interfacial energy of *σ*_1_ = 0.1 J/m^2^ are considered. Thus, the anisotropy parameter of interfacial energy *β*_σ_ = 1.45, and anisotropy feature of interfacial energy equals to *σ*_1_/*σ*_0_ = 1/(1 + *β*_σ_) = 1/2.45 = 0.41. The anisotropic parameters are listed in Table 1.

## 3. Simulation Parameters

### 3.1. Thermodynamic Inputs

**Figure 2:**
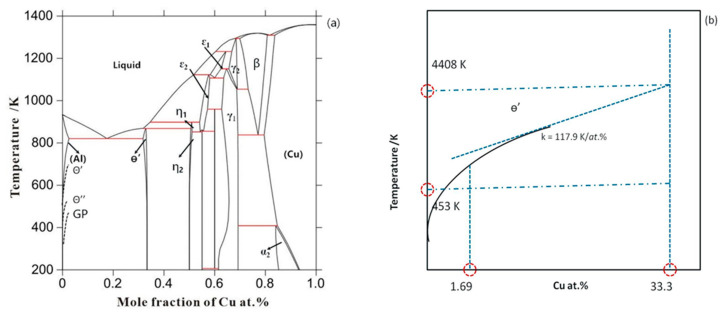
(**a**) Calculated Al-Cu phase diagram based on TCAL5 by means of Thermal-Calc [53]; and (**b**) schematic illustration of the linearization of FCC phase boundary in the phase diagram [55].

The prerequisite for our phase-field simulations is a coupling with the CALPHAD thermodynamic and kinetic databases, which provide realistic chemical driving forces, chemical diffusion potentials and chemical mobilities for the relevant composition and temperature ranges. The calculated Al-Cu phase diagram based on TCAL5 (Thermodynamic database for Al-based alloy) by means of Thermal-Calc [53] is shown in Figure 2a. The metastable solvus curves for GP-zones, θ″ and θ′ precipitates based on literature [54] are also illustrated in the phase diagram. A linearization of the current phase diagram as described in [54] is used to obtain the chemical driving forces. This is schematically shown in Figure 2b. The θ′ phase is treated as stoichiometric phase. For the Al-1.69 at.% Cu alloy at 453 K, the calculated equilibrium phase composition of the θ′ phase is 0.25 at.%, and the calculated equilibrium phase fraction of θ′ is 4.37%.

### 3.2. Other Thermophysical and Numerical Parameters

The summary of the parameters used in the present phase-field simulations are listed in Table 2. The initial Cu concentration is 1.69 at.%. A diffusion coefficient of 1.66 × 10^−20^ m^2^/s was calculated for solute Cu in the fcc-Al matrix at 453 K based on literature data [56]. Large-scale simulation domains were considered for studying ripening with comparable precipitate densities to that of experiments. To find the optimum value of grid spacing ∆*x,* systematic studies were conducted for different values. A sufficient ∆*x* is chosen, such that there is no significant change in the growth kinetic when it is reduced further. The optimum values of time stepping corresponding to the chosen value of ∆*x* is found by observing that there must be no sudden change in the Cu concentration around the precipitate, i.e., a continuous concentration profile.

For simulations of a single precipitate and two precipitates a 100 × 100 × 100 domain with fine grid spacing of ∆*x =* 0.7 nm was considered. The large-scale phase-field simulations are performed in a three-dimensional (3D) domain with 200 × 200 × 200 grid points and ∆*x =* 1.5 nm, to provide a reasonable precipitate number density, but also computational efficiency. Therefore, we have chosen the smallest ∆*x* which allowed simulation of a statistically large enough number of precipitates within the available computational capacity. The interface width is set to 4 grid points. The coherent interfacial energy between Al-matrix and θ′ was treated as 0.1 J/m^2^ and the incoherent one is set as 0.245 J/m^2^ [14,24].

The elastic coefficients of Al-matrix and θ′ precipitates are taken from the literature [14,48], as listed in Table 3. The stress-free eigenstrains of the precipitate phase are $\varepsilon_{11}^{*}$ = $\varepsilon_{22}^{*}$ = 0.00746, $\varepsilon_{33}^{*}$ = −0.051 [14,48]. The boundaries are set to be periodic for both, the phase-field and the concentration field. Moreover, the normal expansion boundary condition is set for elastic stress calculation [46]. For the present work, the plate-like morphology of the θ′ precipitate is due to the comprehensive effects of anisotropic elasticity and interfacial anisotropy. The imposed inhomogeneous and anisotropic elastic stiffness parameters are shown in Table 3. The degree of elastic anisotropy is calculated based on the formula *A* = 2*C*_44_/(*C*_11_-*C*_12_) and the degree of inhomogeneity is calculated by *C*’ =0.5(*C*_11_-*C*_12_) [57].

## 4. Experimental Details

The comparisons between simulation and experimental results are based on our work on an Al-4Cu-1Li-0.25Mn alloy (wt.%) [48,58,59]. After an age hardening treatment, two plate-shaped phases are formed: T_1_ (Al_2_CuLi) and θ′ precipitates, both of which contribute to the strengthening of the alloy. The volume fraction of θ′ is considerably higher than that of T_1_ so the effect of T_1_ phase is neglected here. In addition, as a result of homogenization annealing, 0.1–1 µm size dispersoids of type Al_20_Cu_2_Mn_3_ are formed, which limit grain growth. Details on the alloy processing can be found in Ref. [48]. The initial focus of our experimental work was on the age hardening behavior at shorter ageing times and the microstructure development of the precipitates up to the hardening maximum (17 h/180 °C) [48]. In a follow-up study, we extended on considering longer ageing times up to 1000 h, as well as the effect of external stress during aging were considered [58], with a focus on the thickness growth of the precipitates.

In [58], high-resolution transmission electron microscopy (HRTEM) was used for precise measurement of the thickness and detail structure of precipitates. However, in [48], only conventional TEM (CTEM) was used. The quantitative determination of the microstructure parameters is described in [59]. Since the thickness of the precipitates is used directly in the calculation of the volume fraction of precipitates [59], the results in [48] (CTEM) are subject to greater uncertainty than those in [58] (HRTEM). Therefore, literature values for the thickness and volume fraction of θ′ in binary alloys were also considered in the current study.

**Figure 3:**
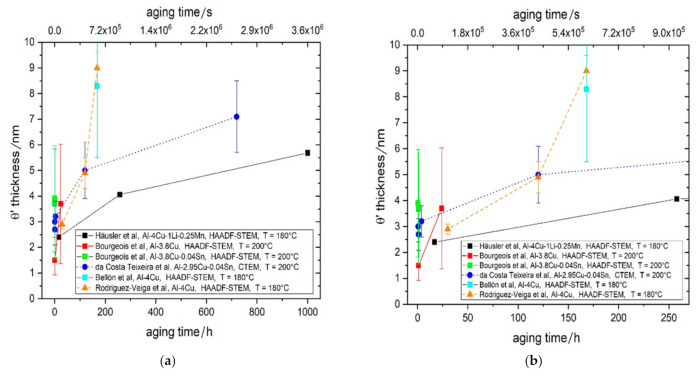
Experimental θ′ thickness data (Häusler et al., Ref [58]) compared to different literature sources (da Costa Teixeira et al., Ref [17], Bourgeois et al., Ref [60], Bellón et al., Ref [62], Rodríguez-Veiga et al., Ref [63]) (**a**) up to 1000 h, (**b**) up to 258 h.

Figure 3 shows a summary of results from different sources for θ′ thickness after aging [17,60,61,62,63]. The data from Häusler et al. [58], Bourgeois et al. [60] and Rodrigues-Veiga [63] are from HR(S)TEM. Note that aging was either at 180 °C or 200 °C. It is striking that the thickness after 17 h/180 °C (black full square, Häusler et al. [58]), Figure 3b and after 24 h/200 °C (red full square, Bourgeois et al. [60]) agree quite well, considering the error bar and differences in time and temperature. Also, the thickness value for 1 h aging of Bourgeois et al. [60] is very reasonable because it is close to the minimum thickness observed for θ′ in general: 2 unit cells in c-direction, i.e., 1.16 nm (see Stobbs and Purdy [64]). Therefore, the mean value for the θ′ thickness was calculated using the histograms given in Figure 5 in [60], which is 1.5 nm +/− 0.6 nm. This value was then used for the volume fraction calculation of θ′ for 1 h using the approach described in [58]. The data for 5 h and 10 h from [48] were not further considered as the thickness data from CTEM is less reliable.

## 5. Results and Discussion

In order to learn how the interfacial anisotropies affect the morphology and growth kinetics of θ′ precipitates, phase-field simulations were performed for single-precipitate, two-precipitates, and multiple-precipitates systems with and without interfacial anisotropy features. Considering that there are several anisotropy scenarios in the present work, the abbreviations in Table 4 will be used in the following where suitable.

### 5.1. Single Precipitate

**Figure 4:**
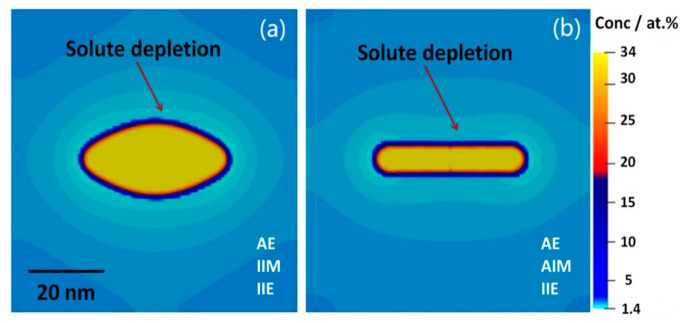
Two-dimensional-cross section of 3D phase-field simulation (concentration field) of the single precipitate in Al-1.69 at.% Cu alloy annealed at 453 K. (**a**) anisotropic elasticity (AE), isotropic interfacial mobility (IIM) and isotropic interfacial energy (IIE) and (**b**) AE, anisotropic interfacial mobility (AIM) and IIE.

Three-dimensional (3D) phase-field simulations were performed with the simulation box size being 100 × 100 × 100. Two-dimensional (2-D) cuts of the concentration field for a single precipitate with isotropic/anisotropic interfacial mobility can be seen in the Figure 4a,b, while keeping isotropic interfacial energy and anisotropic elasticity the same. The light blue areas depict the regions in the matrix around the precipitate that are solute depleted.

With the isotropic mobility, a thick precipitate forms as shown in Figure 4a. In the presence of the anisotropic interface mobility, i.e., the significantly higher mobility at the rim, we obtain a thin, plate-like precipitate. The anisotropic interfacial contribution not only modifies the morphology, but also changes the solute distribution around the precipitate, Figure 4b. The rim of the precipitate grows much wider as compared to the isotropic case, whereas the growth along the vertical direction is suppressed by the low interfacial mobility. The interfacial mobility anisotropy gives a larger aspect ratio (about 3× higher than for the isotropic interfacial mobility), which is closer to the experimentally observed precipitates.

**Figure 5:**
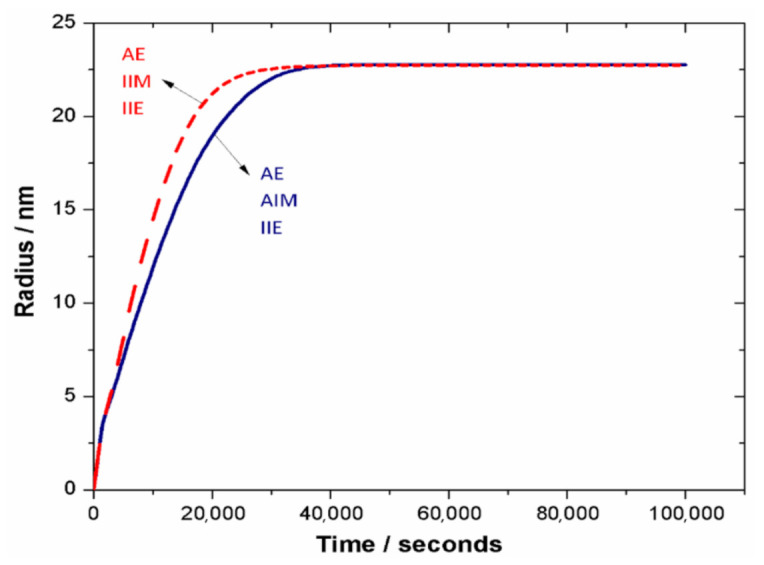
Phase-field simulated equivalent average radius of a single θ′ precipitate vs. time in Al-1.69 at.% Cu alloy annealed at 453 K.

The temporal evolution of the θ′ precipitate is described by its equivalent radius ($R={\left(\frac{3}{4\pi }\sum \phi_{\theta^{\prime }}\;\right)}^{1/3}\Delta x$)), Figure 5. The anisotropic interfacial mobility has reduced the kinetics of the precipitation process at the early growth stage (blue curve). Later, when the equilibrium is reached, same radii (volume fractions) are achieved.

**Figure 6:**
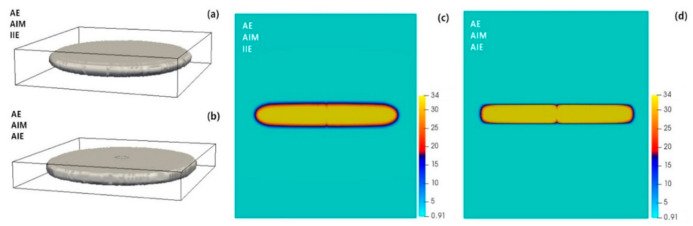
Three-dimensional view and 2D-cross section of a single θ′ precipitate with (**a**,**c**) AE, AIM and IIE conditions and (**b**,**d**) AE, AIM and AIE conditions in Al-1.69 at.% Cu alloy annealed at 453 K for *t* = 100,000 s. Here a 100 × 100 × 100 domain with grid spacing of ∆*x =* 0.7 is used.

The effect of adding anisotropic interfacial energy is compared in Figure 6, while all other parameters were kept the same. With anisotropic interfacial energy, we found that the θ′ precipitate has almost the same thickness but tends to flatten at the rim which produces some points with higher local curvatures (κ) at conjunctions with the faces. This can be understood based on the Gibbs-Thomson effect, i.e., the σκ energy term, where lower interface energy closer to the faces allows for higher curvatures so that the high-energy rims can be more flatten (lower curvature). The results indicate that the effects of interfacial energy anisotropy are rather small, and the aspect ratio of the precipitate is almost unchanged. Instead, the anisotropic interfacial mobility and elasticity play a dominant role, leading to the plate-like morphology of the precipitate, Figure 4.

**Figure 7:**
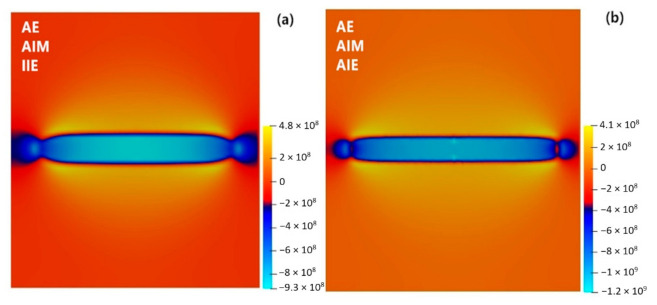
Stress (*σ*_xx_) distribution around the θ′ precipitate with (**a**) AE, AIM and IIE conditions and (**b**) AE, AIM and AIE conditions in Al-1.69 at.% Cu alloy annealed at 453 K for *t* = 100,000 s.

Figure 7 shows the stress (*σ_xx_*) fields. Compression stress inside the precipitate and tension stress around the precipitate are observed which is due to the large negative eigenstrain of the precipitate, Table 2. The anisotropy of the elastic properties results in anisotropic stress fields around the precipitate, i.e., the stress/strain fields around the precipitate greatly change from faces to the rim. The interfacial anisotropies also can affect these stresses by modifying the shape of the precipitate. For instance, due to the flatter plate-like morphology, the tension stress along the surface becomes lower for anisotropic interfacial energy condition, while the compression stress around the rim becomes larger, which is related to the shape changes at the rim of the precipitate.

The stress fields have important implications for the diffusion-controlled growth process as well. The flattening of the faces of the precipitates results in more uniform stresses normal to the precipitate faces. This can assist with the formation of strain-stabilized concentration gradients [7,9]. The stress state at the rim of the θ′ precipitate, however, is very different; A dramatic change in the stress states from faces to rims is obvious, with or without anisotropic interfacial properties. The origin of this is the anisotropy of the elastic properties which cause anisotropic precipitate’s shape and stress/strain fields around it. As a result, the solutal gradients and stress-driven fluxes at the rim and between the faces and rims can change. This has important consequences disturbing the chemo-mechanical effects and the conditions for inverse ripening [1] which is further discussed in Section 5.3.

### 5.2. Two Precipitates

θ′-phase has three different variants because of which the interaction among the precipitates (through both strain and concentration fields) is expected to be far from isotropic. To investigate the behavior of two variants in close proximity to each other, systems of two precipitates with a small inter-precipitate distance were simulated. For the isotropic case, a large matrix around the precipitates (in terms of concentration) is affected by its growth that can be due to the precipitate geometry.

**Figure 8:**
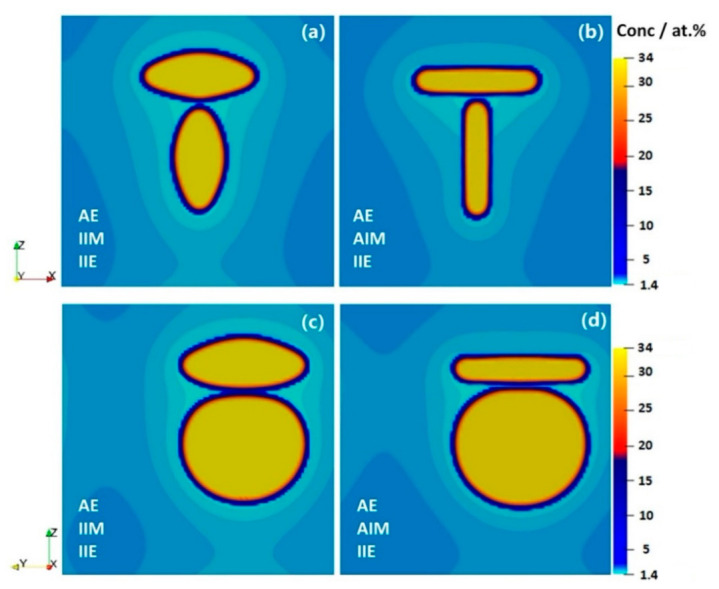
2D-cross section of phase-field simulated concentration fields of two perpendicular precipitates with (**a**,**c**) AE, IIM and IIE conditions and (**b**,**d**) AE, AIM and IIE conditions in Al-1.69 at.% Cu alloy annealed at 453 K for *t* = 15,000 s, along different directions. Here a 100 × 100 × 100 domain with grid spacing of ∆*x =* 0.7 is used.

**Figure 9:**
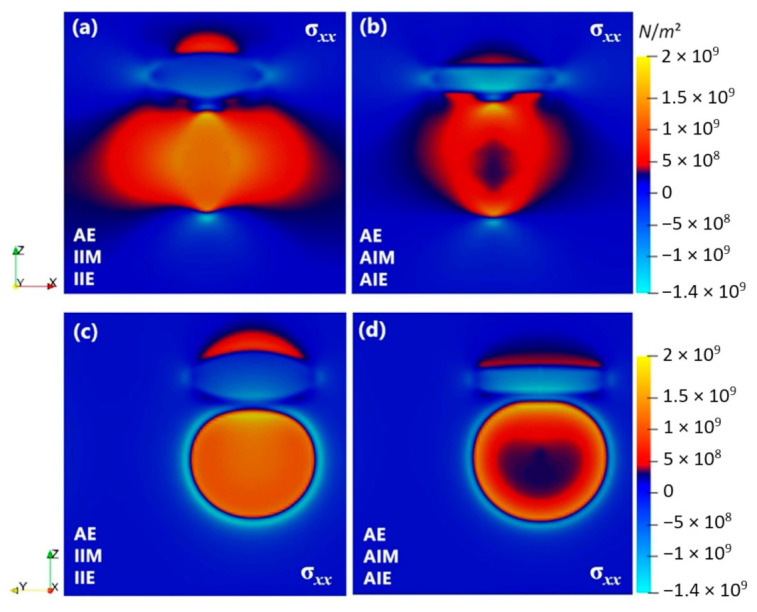
Two-dimensional-cross section of phase-field simulated stress (*σ*_xx_) profiles of two perpendicular precipitates with (**a**,**c**) AE, IIM and IIE conditions and (**b**,**d**) AE, AIM and AIE conditions in Al-1.69 at.% Cu alloy annealed at 453 K for *t* = 15,000 s, along different directions. Here a 100 × 100 × 100 domain with grid spacing of ∆*x =* 0.7 is used.

Figure 8 shows the cross-section concentration maps of evolving two-precipitate systems. The precipitates compete for the solute which depletes near the precipitates and also affect the growth morphology of each other, due to the overlap of their respective stress fields in the matrix, as shown in Figure 9. Compared to the single-precipitate case, the level of stress increases when precipitates are closer to each other. Hence, in addition to the face-to-rim stress changes that can greatly modify the chemo-mechanical features, the interaction between precipitates of various variants magnifies the trend disturbing the possibility of an inverse ripening [1]. It is worth noting that the underlying atomistic structure of interfaces may induce physics far more complex than the current anisotropy consideration that are beyond the scope of the current study.

### 5.3. Effect of Chemo-Mechanical Coupling on θ′ Evolution

**Figure 10:**
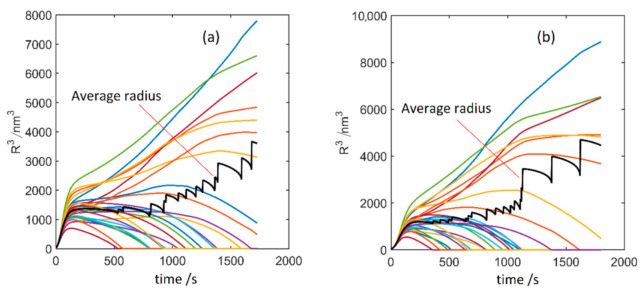
Evolution of θ′ precipitates; (**a**) without; and (**b**) with the chemo-mechanical coupling effect. In contrast to the spherical δ′ precipitates in Al-Li (Figure 3 in Ref. [10]), the coupling has almost no effect on the θ′ ripening. The colored lines associate with the individual θ′ precipitates in the simulation box.

Our results above show that the main source of the anisotropic shape of the θ′ precipitate is the anisotropy of elastic properties, resulting in the anisotropic stress fields around it. These anisotropic stresses can have large impacts on the effect of the chemo-mechanical coupling during the precipitates’ ripening. Figure 10 compares two simulation tests of multiple-precipitates systems with and without the chemo-mechanical coupling. These simulations are done for Δ*x* = 1 nm, *μ* = 3 × 10^−18^ m^4^/Js and an interface width of 5 grid spacing. Randomly distributed nuclei of three different θ′ variants were initially seeded. Further details on these simulations can be found in [65].

We found that the chemo-mechanical coupling has no significant effect on the ripening of the θ′ precipitates. This contrasts with our previous studies, where the composition dependence of elastic constants was shown to dramatically change the course of competitive ripening of δ’ precipitates in Al-Li alloys to inverse ripening, evidenced by in-situ TEM studies [1].

From the TEM investigations on δ’ precipitates in Al-Li system [1], we have found indications that the inverse ripening effect could be extremely sensitive to the stress fields around the precipitates, that can be modified due to the microstructure defects, e.g., grain boundaries, dislocations and free surfaces. The current results confirm the previous observation showing that the elastic anisotropy from the θ′ precipitate’s face to its rim and the interactions among multiple variants of θ′ precipitates disturb the chemo-mechanical effects and therefore a normal ripening is restored. These indicate that the inverse ripening phenomena might be limited to spherical precipitates, such as δ’ phase in Al-Li alloys.

### 5.4. Large Scale Simulation and Comparison to the Experiments

Simulations with a high number of precipitates are required for a complete understanding of the θ′ precipitation kinetics. Based on the precipitate number density 3.0 × 10^−6^ nm^−3^ measured in our experiments, 82 precipitates were seeded into a 300 × 300 × 300 nm^3^ simulation box (with ∆*x =* 1.5 nm) as initial state for the ripening process. We only consider anisotropic interface mobility and elasticity which dominate the precipitation process, as shown above.

**Figure 11:**
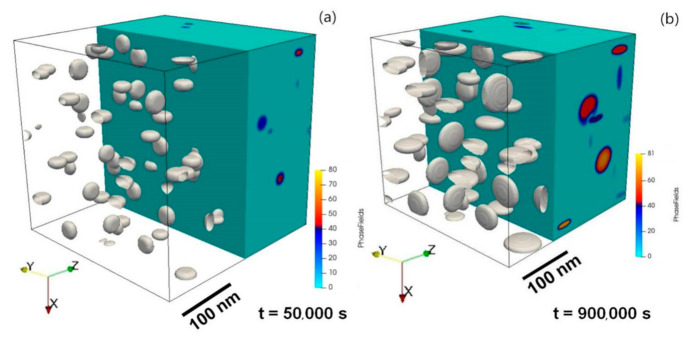
3D large scale phase-field simulated microstructure evolution of θ′ precipitation process in Al-1.69 at.% Cu alloy annealed at 453 K: (**a**) Aging time step = 50,000 s; (**b**) aging time step = 900,000 s.

**Figure 12:**
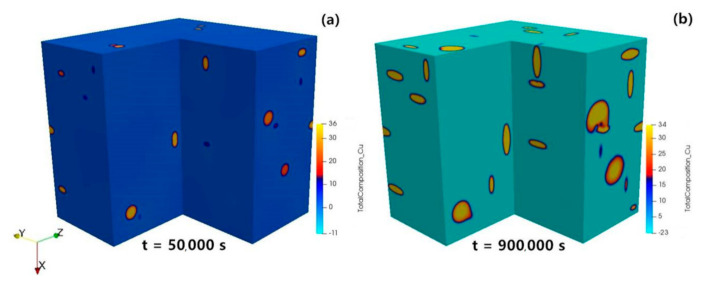
3D large scale phase-field simulated concentration evolution of θ′ precipitation process in Al-1.69 at.% Cu alloy annealed at 453 K: (**a**) Aging time step = 50,000 s; (**b**) aging time step = 900,000 s.

Three different variants of precipitates were randomly positioned for the initial state as seen in Figure 11a, which represent the three possible crystallographic variants/structures observed in the experiment [48]. The evolution of the concentration field during the ripening of θ′ precipitates is shown in Figure 12.

**Figure 13:**
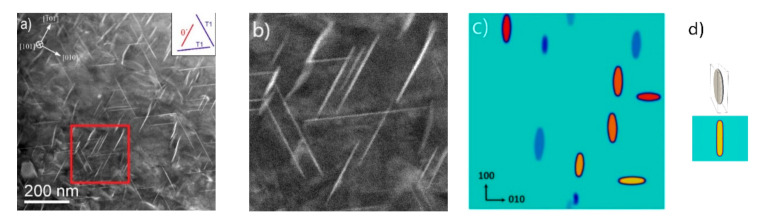
Comparison between the experimental result (Al-4Cu-1Li-0.25Mn alloy, aged 17 h/180 °C; (**a**) overview, (**b**) enlargement, and (**c**) the phase-field simulated morphology of θ′ precipitates in Al-1.69 at.% Cu alloy at 473 K (∆*x =* 1.5 nm) at timestep 90,000 s. Note that only one of the 3 plate shaped precipitate variants represent θ′ (red line in legend (**a**)), while the other two are T_1_. (**d**) The single-precipitate simulation (from Figure 6) with ∆*x =* 0.7 nm is shown for comparing the effect of grid spacing on the precipitate’s aspect ratio.

**Figure 14:**
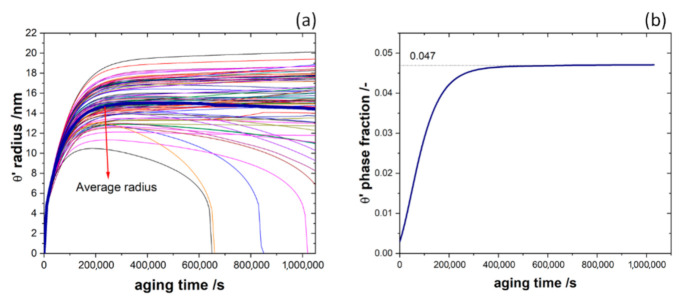
(**a**) Equivalent precipitate radius vs. time for 82 precipitates simulation in Al-1.69 at.% Cu alloy annealed at 453 K for 480,000 s; (**b**) temporal evolution of the precipitates’ phase fraction. The colored lines associate with the individual θ′ precipitates in the simulation box.

The simulated and experimental results are compared in Figure 13. Compared to our previous study [48], the morphology of the precipitates is closer to the experiment. There are still differences which are due to the resolution in the simulation domain. We found that the grid spacing ∆*x* has a major effect on the aspect ratio; the smaller ∆*x* gives more realistic results closer to the experimental observation. The simulation for a single precipitate with 70 × 70 × 70 nm^3^ domain with fine grid spacing of ∆*x =* 0.7 nm is shown in Figure 14d for comparison. To consider such a fine grid spacing in considering large simulation domain with statistically significant number of precipitates requires much larger computational capacity.

**Figure 15:**
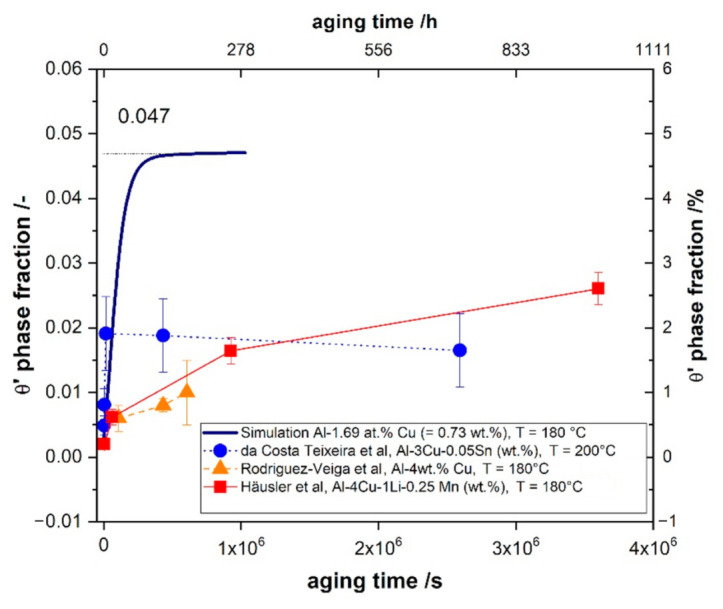
Temporal evolution of the volume fraction of the θ′ phase for 82 precipitates simulation (continuous blue line) in Al-1.69 at.% Cu alloy annealed at 453 K for 480,000 s (cf. Figure 14b) compared with experimental data (da Costa Teixeira et al., Ref [17], Häusler et al., Ref [58], Rodríguez-Veiga et al., Ref [63]).

Figure 14a shows that some smaller precipitates shrink/disappear and competitive ripening is initiated slowly. The average precipitate radius of the 82 precipitates, highlighted by the thicker line, increases over time. A comparison between experimental and simulation results is shown in Figure 15. Here, the volume fraction of the θ′ phase from both simulation (continuous blue line) and experiment is plotted vs. time. The early growth kinetics agree well but the evolution of the precipitates in the experiment is suppressed in the later stages. The red full squares represent our experimental data [17,58,63] with a newly calculated value for 1 h as described in Section 4. Our experimental data (red curve) agree well with data from Rodriguez-Veiga et al. [63] at the same aging temperature (orange triangles). Data from da Costa Teixeira et al. [17] (blue full circles) shows a higher fraction for shorter aging times, which might be due to the higher aging temperature of 200 °C.

## 6. Summary and Outlook

A series of multi-phase-field simulations with anisotropic interfacial energy and mobility effects were performed to investigate the plate-like metastable strengthening phase θ′ (Al_2_Cu) in an Al-1.69 at.% Cu alloy at 453 K during the growth and ripening process. We found that the main shape-controlling factors are the elastic anisotropy of the precipitate and the anisotropic interfacial mobility that favor the growth along the rim of the precipitate and increase its aspect ratio. In contrast, the interface energy anisotropy shows little effects only modifying the shape of the precipitate’s rim. Experimental results from our previous work were reassessed, combined and supplemented with new data from the literature based on precise thickness measurements of the precipitates. There is a good agreement with quantitative results from other works. The incorporation of the anisotropy effects brings our phase-field simulation results closer to our experimental observations.

In terms of the stress fields, the elastic anisotropy of the precipitate plays the central role resulting in anisotropic stress fields around the precipitates. The interfacial anisotropy features further enhance the anisotropy of the stress fields around the θ′ precipitate. In addition, the interaction among the θ′ precipitates of various variants can dramatically influence the stress fields around the precipitates and their evolution. The stress fields play an important role as it governs the effects of chemo-mechanical coupling on the ripening process. We have found that in the presence of such anisotropic stress fields around the precipitates, the chemo-mechanically-driven inverse ripening, recently reported for the spherical δ’ precipitates in Al-Li alloys [1], cannot be active in the case of plate-like θ′ precipitates.

As an outlook, we suggest exploring the role of chemo-mechanical coupling effects on the formation of more spherical precipitates. The core-shell spherical precipitates, such as Al_3_(Li, Sc) in the Al-Li-Sc alloy system (with a Li-rich ‘shell’ forming around an Sc-rich spherical ‘core’) [11] could be particularly interesting. Formation of the nanometer size shell layer around the core, where the overlap between the diffusion gradients and stress fields are significant and stresses can be large, can be influenced by the chemo-mechanical coupling. Here the atomic size difference between the Li and Sc solute atoms can produce chemo-mechanically driven solute fluxes contributing to the core-shell formation.

## Figures and Tables

**Table 1:** Anisotropy parameters of interfacial energy and interfacial mobility [24,49].

Property	$\mu_{0}/\sigma_{0}$	*φ_0_*	*β*
Interfacial mobility *μ*/cm^4^/Js	1.0 × 10^−19^	π/10,000	2000
Interfacial energy *σ*/J/m^2^	0.245	π/10,000	1.45

**Table 2:** Summary of the parameters in the present phase-field simulations.

Variable	Symbol	Value/Unit	Ref.
Interfacial width	*η*	2.8 × 10^−9^–6 × 10^−9^ m	-
Grid spacing	Δ*x*	0.7 × 10^−9^–1.5 × 10^−9^ m	-
Time step	d*t*	1 s	-
Interfacial energy	*σ*_0_	0.245 J/m^2^	[24]
Interfacial mobility	*μ*_0_	1 × 10^−19^ m^4^/Js	-
Diffusivity of fcc Al	*D*	1.66 × 10^−20^ m^2^/s	[56]
Thermodynamics	Δ*g*	Database	[54]
Eigenstrain	${\bar{\varepsilon }}_{}^{*}$	0.00746, 0.00746, −0.051	[14]

**Table 3:** Elastic constants of the matrix and precipitate phases.

Phase	C_11_/GPa	C_12_/GPa	C_44_/GPa	A/-	C’/-
fcc Al matrix	107.07	63.08	28.52	1.297	21.995
θ′	190	80	90	1.636	55

**Table 4:** Isotropy conditions of the simulations abbreviated.

Condition	Anisotropic Elasticity (Always Included)	Anisotropic Interfacial Mobility	Isotropic Interfacial Mobility	Anisotropic Interfacial Energy	Isotropic Interfacial Energy
Abbreviation	AE	AIM	IIM	AIE	IIE

## Data Availability

The data of these findings cannot be shared at this time as the data also form part of an ongoing study.

## References

[1] Park J., Darvishi Kamachali R., Kim S.-D., Kim S.-H., Oh C.-S., Schwarze C., Steinbach I. (2019). First Evidence for Mechanism of Inverse Ripening from In-situ TEM and Phase-Field Study of δ′ Precipitation in an Al-Li Alloy. Sci. Rep..

[2] Larché F.C., Cahn J.W. (1985). Overview no. 41 the interactions of composition and stress in crystalline solids. Acta Metall. Mater..

[3] Larché F.C., Cahn J.W. (1978). A nonlinear theory of thermochemical equilibrium of solids under stress. Acta Metall. Mater..

[4] Fratzl P., Penrose O., Lebowitz J.L. (1999). Modeling of Phase Separation in Alloys with Coherent Elastic Misfit. J. Stat. Phys..

[5] Shi S., Markmann J., Weissmüller J. (2018). Verifying Larché–Cahn elasticity, a milestone of 20th-century thermodynamics. Proc. Natl. Acad. Sci. USA.

[6] Singh A., Pal S. (2020). Coupled chemo-mechanical modeling of fracture in polycrystalline cathode for lithium-ion battery. Int. J. Plast..

[7] Darvishi Kamachali R., Borukhovich E., Shchyglo O., Steinbach I. (2013). Solutal gradients in strained equilibrium. Philos. Mag. Lett..

[8] Darvishi Kamachali R., Borukhovich E., Hatcher N., Steinbach I. (2014). DFT-supported phase-field study on the effect of mechanically driven fluxes in Ni_4_Ti_3_ precipitation. Model. Simul. Mater. Sci. Eng..

[9] Darvishi Kamachali R., Schwarze C. (2017). Inverse Ripening and Rearrangement of Precipitates under Chemomechanical Coupling. Comput. Mater. Sci..

[10] Schwarze C., Gupta A., Hickel T., Darvishi Kamachali R. (2017). Phase-field study of ripening and rearrangement of precipitates under chemomechanical coupling. Phys. Rev. B.

[11] Radmilovic V., Ophus C., Marquis E.A., Rossell M.D., Tolley A., Gautam A., Asta M., Dahmen U. (2011). Highly monodisperse core–shell particles created by solid-state reactions. Nat. Mater..

[12] Polmear I.J. (2006). Light Alloys—From Traditional Alloys to Nanocrystals.

[13] Prasad N.E., Gokhale A.A., Wanhill R.J.H. (2014). Aluminum-Lithium-Alloys. Processing, Properties and Applications.

[14] Vaithyanathan V., Wolverton C., Chen L.Q. (2004). Multiscale modeling of θ′ precipitation in Al-Cu binary alloys. Acta Mater..

[15] Ringer S.P., Hono K. (2000). Microstructural Evolution and Age Hardening in Aluminium Alloys: Atom Probe Field-Ion Microscopy and Transmission Electron Microscopy Studies. Mater. Charact..

[16] Phillips V.A. (1973). Lattice resolution measurement of strain fields at guinier-preston zones in Al-3.0 % Cu. Acta Metall..

[17] Da Costa Teixeira J., Cram D.G., Bourgeois L., Bastow T.J., Hill A.J., Hutchinson C.R. (2008). On the strengthening response of aluminum alloys containing shear-resistant plate-shaped precipitates. Acta Mater..

[18] Nie J.F., Muddle B.C. (1998). Microstructural design of high-strength aluminum alloys. J. Phase Equilib..

[19] Wolverton C. (1999). First-principles prediction of equilibrium precipitate shapes in aluminium-copper alloys. Philos. Mag. Lett..

[20] Huang Y., Robson J.D., Prangnell P.B. (2009). The formation of nanograin structures and accelerated room-temperature theta precipitation in a severely deformed Al-4 wt.% Cu alloy. Acta Mater..

[21] Vaughan D. (1968). Grain boundary precipitation in an aluminium-copper alloy. Acta Metall..

[22] Hillert M. (1965). On the theory of normal and abnormal grain growth. Acta Metall..

[23] Jiang H., Faulkner R.G. (1995). Modelling of grain boundary segregation, precipitation and precipitate free zones of high strength aluminium alloys—I. The model. Acta Mater..

[24] Khan I.N., Starink M.J., Yan J.L. (2008). A model for precipitation kinetics and strengthening in Al–Cu–Mg alloys. Mater. Sci. Eng. A.

[25] Campisano S.U., Costanzo E., Scaccianoce F., Cristofolini R. (1978). Growth kinetics of the phase in aluminium-copper thin film bilayers. Thin Solid Films.

[26] Jena A.K., Gupta A.K., Chaturvedi M.C. (1989). A differential scanning calorimetric investigation of precipitation kinetics in the Al-1.53 wt.% Cu-0.79 wt.% Mg alloy. Acta Metall..

[27] Holzeri J.C., Kelton K.F. (1991). Kinetics of the amorphous to icosahedral phase transformation in Al-Cu-V alloys. Acta Metall Mater..

[28] Huang B.P., Zheng Z.Q. (1998). Independent and combined roles of trace Mg and Ag additions in properties precipitation process and precipitation kinetics of Al–Cu–Li–(Mg)–(Ag)–Zr–Ti alloys. Acta Mater..

[29] Larouche D. (2017). Mixed mode growth of an ellipsoidal precipitate: Analytical solution for shape preserving growth in the quasi-stationary regime. Acta Mater..

[30] Heugue P., Larouche D., Breton F., Martinez R., Chen X.G. (2019). Evaluation of the Growth Kinetics of θ’ and θ-Al_2_Cu Precipitates in a Binary Al-3.5 wt.% Cu Alloy. Metall. Mater. Trans. A.

[31] Deschamps A., Brechet Y. (1998). Influence of predeformation and aging of an Al–Zn–Mg alloy—II. Modeling of precipitation kinetics and yield stress. Acta Mater..

[32] Mishin Y., Asta M., Li J. (2010). Atomistic modeling of interfaces and their impact on microstructure and properties. Acta Mater..

[33] Wang J., Wolverton C., Muller S., Liu Z.K., Chen L.Q. (2005). First-principles growth kinetics and morphological evolution of Cu nanoscale particles in Al. Acta Mater..

[34] Schleifer F., Fleck M., Holzinger M., Lin Y.-Y., Glatzel U., Tin S., Hardy M., Clews J., Cormier J., Feng Q., Marcin J., O’Brien C., Suzuki A. (2020). Phase-field modeling of γ′ and γ′′ precipitate size evolution during heat treatment of Ni-base superalloys. Superalloys 2020.

[35] Schwarze C., Darvishi Kamachali R., Kühbach M., Mießen C., Tegeler M., Barrales-Mora L., Steinbach I., Gottstein G. (2018). Computationally Efficient Phase-field Simulation Studies Using RVE Sampling and Statistical Analysis. Comput. Mater. Sci..

[36] Schleifer F., Holzinger M., Lin Y.-Y., Glatzel U., Fleck M. (2020). Phase-field modeling of γ/γ″ microstructure formation in Ni-based superalloys with high γ″ volume fraction. Intermetallics.

[37] Li D.Y., Chen L.Q. (1998). Computer simulation of stress-oriented nucleation and growth of θ′ precipitates in Al–Cu alloys. Acta Mater..

[38] Vaithyanathan V., Wolverton C., Chen L.Q. (2002). Multiscale modeling of precipitate microstructure evolution. Phys. Rev. Lett..

[39] Darvishi Kamachali R., Schwarze C., Lin M., Diehl M., Shanthraj P., Prahl U., Steinbach I., Raabe D. (2018). Numerical Benchmark of Phase-Field Simulations with Elastic Strains: Precipitation in the Presence of Chemo-Mechanical Coupling. Comput. Mater. Sci..

[40] Tegeler M., Shchyglo O., Darvishi Kamachali R., Monas A., Steinbach I., Sutmann G. (2017). Parallel multiphase field simulations with OpenPhase. Comput. Phys. Commun..

[41] Steinbach I. (2009). Phase-field models in materials science. Model. Simul. Mater. Sci. Eng..

[42] Steinbach I. (2013). Phase-field model for microstructure evolution at the mesoscopic scale. Ann. Rev. Mater. Res..

[43] Steinbach I., Pezzolla F., Nestler B., Seeßelberg M., Prieler R., Schmitz G.J., Rezende J.L.L. (1996). A phase field concept for multiphase systems. Phys. D.

[44] Eiken J., Böttger B., Steinbach I. (2006). Multiphase-field approach for multicomponent alloys with extrapolation scheme for numerical application. Phys. Rev. E.

[45] Anderson J.O., Helander T., Höglund L., Shi P., Sundman B. (2002). Thermo-Calc & DICTRA, Computational Tools for Materials Science. Calphad.

[46] Steinbach I., Apel M. (2006). Multi phase field model for solid state transformation with elastic strain. Phys. D.

[47] Borgenstam A., Höglund L., Agren J., Engström A. (2000). DICTRA, a tool for simulation of diffusional transformations in alloys. J. Phase Equilib..

[48] Häusler I., Schwarze C., Umer Bilal M., Valencia Ramirez D., Hetaba W., Darvishi Kamachali R., Skrotzki B. (2017). Precipitation of T_1_ and θ′ Phase in Al-4Cu-1Li-0.25Mn During Age Hardening: Microstructural Investigation and Phase-Field Simulation. Materials.

[49] Ji Y., Ghaffari B., Li M., Chen L.-Q. (2018). Phase-field modeling of θ′ precipitation kinetics in 319 aluminum alloys. Comput. Mater. Sci..

[50] Debierre J.-M., Karma A., Celestini F., Guérin R. (2003). Phase-field approach for faceted solidification. Phys. Rev. E.

[51] Eggleston J.J., McFadden G.B., Voorhees P.W. (2001). A phase-field model for highly anisotropic interfacial energy. Phys. D.

[52] Fleck M., Federmann H., Pogorelov E. (2018). Phase-field modeling of Li-insertion kinetics in single LiFePO4-nano-particles for rechargeable Li-ion battery application. Comput. Mater. Sci..

[53] Thermo-Calc-Software. https://www.thermocalc.com/media/19849/tcal5_extended_info.pdf.

[54] Murray J.L. (1985). The aluminium-copper system. Int. Mater. Rev..

[55] Fuller C.B., Murray J.L., Seidman D.N. (2005). Temporal evolution of the nanostructure of Al(Sc,Zr) alloys: Part I—Chemical compositions of Al_3_(Sc_1−x_Zr_x_) precipitates. Acta Mater..

[56] Liu D., Zhang L., Du Y., Xu H., Liu S., Liu L. (2009). Assessment of atomic mobilities of Al and Cu in fcc Al-Cu alloys. Calphad.

[57] Vallin J., Mongy M., Salama K., Beckman O. (1964). Elastic constants of aluminum. J. Appl. Phys..

[58] Häusler I., Darvishi Kamachali R., Hetaba W., Skrotzki B. (2019). Thickening of T_1_ Precipitates during Aging of a High Purity Al–4Cu–1Li–0.25Mn Alloy. Materials.

[59] Häusler I. (2017). Determination of volume fraction of discrete oriented circlar disc-shaped preicpitates in the transmission mode. Pract. Metallogr..

[60] Bourgeois L., Dwyer C., Weyland M., Nie J.-F., Muddle B.C. (2012). The magic thicknesses of θ′ precipitates in Sn-microalloyed Al-Cu. Acta Mater..

[61] Bourgeois L., Dwyer C., Weyland M., Nie J.-F., Muddle B.C. (2011). Structure and energetics of the coherent interface between the θ′ precipitate phase and aluminium in Al-Cu. Acta Mater..

[62] Bellón B., Haouala S., Llorca J. (2020). An analysis of the influence of the precipitate type on the mechanical behavior of Al-Cu alloys by means of micropillar compression tests. Acta Mater..

[63] Rodríguez-Veiga A., Bellón B., Papadimitriou I., Esteban-Manzanares G., Sabirov I., Llorca J. (2018). A multidisciplinary approach to study precipitation kinetics and hardening in an Al-4Cu (wt.%) alloy. J. Alloy. Compd..

[64] Stobbs W.M., Purdy G.R. (1978). The Elastic Accommodation of Semicoherent θ′ in Al-4 wt.% Cu Alloy. Acta Metall..

[65] Umer Bilal M. (2016). Growth Kinetics and Coherency Loss from θ′ to θ in Al-Cu Alloy: A Phase-Field Study. Master’s Thesis.

